# Uptake and Use of Care Companion, a Web-Based Information Resource for Supporting Informal Carers of Older People: Mixed Methods Study

**DOI:** 10.2196/41185

**Published:** 2023-09-21

**Authors:** Jeremy Dale, Veronica Nanton, Theresa Day, Patricia Apenteng, Celia Janine Bernstein, Gillian Grason Smith, Peter Strong, Rob Procter

**Affiliations:** 1 Academic Primary Care Unit Warwick Medical School University of Warwick Coventry United Kingdom; 2 Institute of Applied Health Research University of Birmingham Birmingham United Kingdom; 3 Carers4Carers Warwickshire United Kingdom; 4 Centre for Complexity Science University of Warwick Coventry United Kingdom; 5 The Alan Turing Institute London United Kingdom; 6 Department of Computer Science University of Warwick Coventry United Kingdom

**Keywords:** informal carers, information technology, internet, information needs, mixed methods evaluation, Reach, Effectiveness, Adoption, Implementation, and Maintenance, RE-AIM, mobile phone

## Abstract

**Background:**

Informal carers play a major role in supporting relatives and friends who are sick, disabled, or frail. Access to information, guidance, and support that are relevant to the lives and circumstances of carers is critical to carers feeling supported in their role. When unmet, this need is known to adversely affect carer resilience and well-being. To address this problem, Care Companion was co-designed with current and former carers and stakeholders as a free-to-use, web-based resource to provide access to a broad range of tailored information, including links to local and national resources.

**Objective:**

This study aimed to investigate the real-world uptake and use of Care Companion in 1 region of England (with known carer population of approximately 100,000), with local health, community, and social care teams being asked to actively promote its use.

**Methods:**

The study had a convergent parallel, mixed methods design and drew on the RE-AIM (Reach, Effectiveness, Adoption, Implementation, and Maintenance) framework. Data included metrics from carers’ use of Care Companion, surveys completed by users recruited through general practice, and interviews with carers and health and social care providers regarding their views about Care Companion and their response to it. Quantitative data were analyzed using descriptive statistics. Interview data were analyzed thematically and synthesized to create overarching themes. The qualitative findings were used for in-depth exploration and interpretation of quantitative results.

**Results:**

Despite awareness-raising activities by relevant health, social care, and community organizations, there was limited uptake with only 556 carers (0.87% of the known carer population of 100,000) registering to use Care Companion in total, with median of 2 (mean 7.2; mode 2) visits per registered user. Interviews with carers (n=29) and stakeholders (n=12) identified 7 key themes that influenced registration, use, and perceived value: stakeholders’ signposting of carers to Care Companion, expectations about Care Companion, activity levels and conflicting priorities, experience of using Care Companion, relevance to personal circumstances, social isolation and networks, and experience with digital technology. Although many interviewed carers felt that it was potentially useful, few considered it as being of direct relevance to their own circumstances. For some, concerns about social isolation and lack of hands-on support were more pressing issues than the need for information.

**Conclusions:**

The gap between the enthusiastic views expressed by carers during Care Companion’s co-design and the subsequent low level of uptake and user experience observed in this evaluation suggests that the co-design process may have lacked a sufficiently diverse set of viewpoints. Numerous factors were identified as contributing to Care Companion’s level of use, some of which might have been anticipated during its co-design. More emphasis on the development and implementation, including continuing co-design support after deployment, may have supported increased use.

## Introduction

### Background

Informal carers (in this paper, referred to as carers but also known as family caregivers or unpaid carers) provide a substantial amount of support and care to adult family and friends who live with disability and declining health. In the United Kingdom, it has been estimated that there are approximately 10.6 million carers, and during 2020, the first year of the COVID-19 pandemic, they provided an estimated £193 billion (US $243 billion) of care [1,2]. Carers experience significant personal costs in terms of their own health and overall well-being, with many failing to receive the support that they require. This reflects the time-consuming, emotionally and physically exhausting, and often multifaceted caring activities associated with the complex needs of the care recipient [2]. The lack of support for carers is a contributory factor that may limit the capacity to continue caring [3]. With the increasing pressures on both health services and residential and community-based social care [4], there is a need for better ways of providing carer support [5].

In the United Kingdom, the Care Act (2014) recognizes the importance of information and guidance for developing and maintaining carers’ skills and resilience [6]. However, many carers describe a lack of awareness about and access to information resources that are relevant to their changing needs and circumstances [2,7]. They often describe struggling to access and gain help from health and care systems that are difficult to navigate [7]. Reviews of in-person interventions that provide information and advice for carers of people with dementia have found varied results, but there is some evidence of benefit in alleviating caregiver symptoms of depression [8]. However, only a small minority of carers access in-person carer support services, in part owing to the difficulty of leaving the care recipient [9,10].

The internet provides ubiquitous access to information and advice, but it can feel impersonal, difficult to navigate, and unresponsive to individual circumstances [11]. It may be difficult to identify reliable, relevant sources of information [2]. Well-recognized barriers to carers’ use of the internet for information include health and IT literacy, emotional strain, intensity of caring, and financial hardship [12]. Overall, 20% of carers aged >64 years (compared with 10% of young carers) feel that a lack of digital skills hinders their ability to use digital technology [2].

Although information about support services has traditionally been provided in paper format, there is evidence that many carers now prefer to receive information via web-based sources and that this can lead to improved well-being [5]. Compared with face-to-face services, internet-based support interventions are likely to be relatively low cost and potentially more readily tailored to individual needs and hence experienced as useful [13,14]. Their availability 24/7 can also help address the social isolation associated with caring [15]; several systematic reviews of internet-based supportive interventions for carers have reported their potential usefulness and impact on psychological outcomes [5,14-18]. However, many of these studies are small-scale, pilot studies, and the overall evidence base for internet-based interventions for carers remains limited [12].

In this paper, we have reported an evaluation of the real-world uptake and use of Care Companion, when made freely available in 1 region of England, including how registered users and local stakeholder organizations perceived its usefulness. As described in the following section, Care Companion is a web-based information resource that was co-designed to address the need for personalized information for carers, as described in previous *JMIR Aging* publications [19,20].

### Care Companion

Care Companion was co-designed using a *person-based* approach that incorporated the perspectives of carers by synthesizing evidence from the research and policy literature, with active input from carers and stakeholders throughout the developmental process [21]. It was aimed at addressing 4 key challenges: burden of care, lack of knowledge, enhancing self-efficacy, and lack of time [19]. A panel of 5 carers recruited from local support groups provided detailed input regarding the design of its features and content, reflecting their first-hand experience of carers’ needs, and input from a stakeholder group (representatives from local health service commissioning organizations, public health, social care, health providers, third sector, and voluntary organizations) allowed the incorporation of provider and policy perspectives [19].

Care Companion is underpinned by a biopsychosocial model that covers 5 independent domains (extending social assets, strengthening psychological resources, ensuring timely availability of key external resources, maintaining physical health, and safeguarding quality of life), targeted to strengthen carer resilience and coping [22]. A key feature is a rigorously curated library of trusted sources of web-based information and guidance on the broad range of issues that are relevant to the challenges that carers face in their caring roles [19]. Users are encouraged to regularly update their profile with information about their own and their care recipient’s needs and circumstances. Care Companion draws on these data to filter information that is most likely to be of relevance to the carer and care recipient’s circumstances.

Care Companion was launched in June 2018 as a free-to-use resource for people with adult caring responsibilities, tailored to the Coventry and Warwickshire subregion of the West Midlands, England. Its launch was included as part of the local government’s Carers’ Strategy and had the support of local Members of Parliament; local government policy makers and politicians; and key health, social care, and third-sector stakeholder organizations. Over the following 3 years, a broad range of local promotional activities were undertaken with health, social care, and community groups to encourage carer uptake. Alongside this, Care Companion was regularly updated with new content, and its functionality was improved in light of user feedback. From 2020, this included the addition of up-to-date COVID-19–related guidance and locally available support, including information about vaccines and local testing services.

## Methods

### Overview

The study design initially drew on the RE-AIM (Reach, Effectiveness, Adoption, Implementation, and Maintenance) framework [23], an approach that assesses how public health interventions translate to real-world settings, using a convergent parallel, mixed methods approach [24]. The RE-AIM framework requires both qualitative and quantitative methods to understand its dimensions. We planned to explore how the characteristics of registered users compared with those of the wide population of carers within the study setting and how patterns of use were associated with mental health, well-being, and carer resilience to investigate effectiveness. Using qualitative methods, we also intended to explore factors affecting the reach, adoption, implementation, and maintenance of use of Care Companion from carer and stakeholder perspectives. However, the quantitative study was scaled back once it became evident that the level of use of Care Companion was insufficient to support meaningful data analysis. Instead, we focused data collection and analysis on data from Care Companion’s user profiles and use histories; carer surveys; and interviews with carers and stakeholders relevant to consideration of the uptake, adoption, and ongoing use of Care Companion.

### Setting

The study was conducted in Coventry and Warwickshire (a mixed urban and rural area with a total population of 963,173). According to the 2011 census, 66% of Coventry’s population and 93% of Warwickshire’s population are of White ethnic background, and approximately 100,000 people identified themselves as carers [25].

### Carers’ Panel

A panel of carers (chaired by GGS) was recruited from carers’ groups in the study area to support the ongoing development, refinement, and implementation of Care Companion and the design and conduct of the study. The panel commented on all carer-facing research materials and contributed to data analysis and interpretation of findings.

### Participants

The following three groups were included:

1. The study included carers who had registered as users of Care Companion before October 2020 (hereafter, referred to as group 1), following local public-facing and stakeholder organization promotional activity, described previously. They were informed about the study via email and notifications from Care Companion and invited to download participant information and complete a web-based consent form. The consent form included confirming that they had read and understood the participant information. Contact details were also provided, so that participants could ask any questions they had about the study.
2. The study included carers who registered to use Care Companion following signposting from their general practitioner (GP; hereafter, referred to as group 2). Between January 2021 and March 2021, a total of 14 general practices in geographically diverse (rural, urban, and semiurban) areas identified eligible individuals through their registers of patients’ carers. Carers were excluded according to the following criteria:
  - Carer or care recipient aged <18 years
  - Not residing in Coventry or Warwickshire
  - Unable to understand written English or provide informed consent
  - Care recipient known to be acutely ill (eg, currently in hospital) or in the last few weeks of life
  Eligible carers were contacted either via SMS text message or mail, depending on the preference of each practice, and informed about Care Companion and the opportunity to participate in the study. SMS text messages contained links to web-based participant information and consent forms. Mailed letters included the participant information leaflet and a Freepost expression of interest reply slip; on its receipt, a member of the study team made contact to provide access to the consent form. Consented participants were asked to register with Care Companion and, if needed, were offered guidance on the registration process from a member of the research team.
3. The study also included local stakeholders. A wide range of individuals and organizations that provide services for carers, including representatives of charities, local authorities, health and social care commissioning organizations, and people working in the community such as librarians were encouraged to promote the use of Care Companion to their clients, patients, or members. They were contacted directly and via existing networks, newsletters, and phone calls. The same organizations were invited to consider participating in the evaluation, and those that expressed interest were provided with more detailed information about the study and followed up by the research team.

### Data Collection

Data for the study were collected in the following ways.

#### Routine Data About the Use of Care Companion and Associated Materials

Data from the web hosting service were downloaded to provide anonymous information about individual user’s visits to Care Companion, including visit duration and webpages accessed. In addition, data about the opening and use of web-based user guides, videos, email notifications aimed at new users, and monthly Care Companion newsletters were downloaded.

#### Carer Experiences of Care Companion

Carers (both group-1 and group-2 participants) were invited to participate in a semistructured phone interview. Topics included previous digital experience, views about and experiences of caring, motivation to use Care Companion, factors influencing their level of use, and intentions for future use.

In addition, participants recruited via general practice (group 2) were also asked to complete a web-based baseline survey to collect sociodemographic information and a follow-up survey (4-6 months after registration) covering the use of Care Companion, perceived barriers and facilitators, and a free-text space for further comments. Although a similar set of surveys was planned for carers who had registered with Care Companion directly (group 1), owing to the very low response rate to invitations to participate, we decided not to proceed with the second round.

#### Stakeholder Views About Care Companion

Stakeholders were invited to participate in a semistructured phone interview.

The recruitment of stakeholders occurred over a 12-month period, with approximately 350 organizations targeted initially. These covered a range of geographical areas and organization types identified through existing contacts, internet searches, recommendations from other groups, social media searches, and suggestions from the carer’s panel. They included health-related organizations such as general practices; hospital-based teams; hospices; charities; and community-based groups including faith groups, support groups, community networks, and library services. Stakeholders were approached via email, phone calls, Facebook (Meta Platforms, Inc), or Teams (Microsoft Corp) or Zoom (Zoom Video Communications, Inc) meetings.

Of the those contacted, 52 (approximately 15% of the 350 organization) expressed interest and received further information via email, information leaflets, or meetings and were invited to participate in an interview.

The topic guide explored awareness and views about Care Companion; how they had promoted Care Companion to potential users; and perceptions about factors affecting its adoption, use, and relevance.

### Data Analysis

#### Web Analytics

User logs were analyzed to identify the number of visits per user, number of actions performed by each user, and total time spent on Care Companion.

#### Survey Data

Descriptive statistics were produced using SPSS (IBM Corp) [26].

#### Interview Data

All interviews were digitally recorded and transcribed verbatim. Transcripts were entered into NVivo (QSR International) [27]. The interview data for the 3 groups of participants were coded separately by team members, overseen by VN, according to the steps proposed by Braun and Clarke [28]: familiarization with the data, generating initial codes, searching for themes, reviewing themes, and defining and naming themes. Themes were reviewed and discussed with all members of the project team and presented to the study’s carers’ panel. Further codes were generated to reflect the feedback from these sessions and applied to the data informing further thematic development.

#### Convergent Analysis

To identify and explore similarities and differences between the findings emerging from the quantitative and qualitative data analyses, we triangulated the themes identified in the analysis of the patient and stakeholder qualitative data sets and mapped these against the quantitative findings [29]. We then developed thematic categories that provide a representation of the whole data set to support the understanding of the factors that appear to influence the uptake and use of Care Companion.

### Data Interpretation

Regular meetings were conducted with the carers’ panel and a stakeholder panel to support the interpretation of findings and their implications. A workshop to gain further input was conducted with local health care, social care, and third-sector organizations, together with members of the study’s carers’ panel at the end of the study.

### Ethical Considerations

The study received ethics approval from National Health Service (ID 271605; West Midlands–Edgbaston research ethics committee). All eligible individuals were provided with an information leaflet and consent form to be completed before their participation in the study. Consent was confirmed at the time of interview. Participants had the opportunity to withdraw from the study at any stage of data collection. The information leaflet explained that all study data would be deidentified to ensure the anonymity of participants. Participants did not receive any incentive or payment.

## Results

### Care Companion Users and Study Participants

By October 2020, there were 476 registered users of Care Companion (0.74% of the registered carer population of 100,000 in the catchment area). Between January 2021 and March 2021, a further 80 (3.8% of the 2105 invited to participate in the study) by general practices registered with Care Companion, giving a total of 556 users (0.87% of the registered carer population of 100,000).

Overall, 62 different care recipient conditions were recorded in the user profiles; the most frequent were Alzheimer disease and other dementias (188/556, 33.8% of the profiles), anxiety (146/556, 26.3%), depression (120/556, 21.6%), osteoarthritis (104/556, 18.7%), type 2 diabetes (76/556, 13.6%), and urinary incontinence (68/556, 12.2%).

### Carer Interviews and Surveys

In total, 60 carers expressed interest in being interviewed, and 29 (48%) consented and were interviewed; this comprised 67% (10/15) from group 1 and 42% (19/45) from group 2, with a range of characteristics (Table 1). They had experience of caring that ranged from 2.5 to 30 years in duration.

**Table 1:** Characteristics of interview participants.

Characteristics			Group-1 carers, n (%)		Group-2 carers, n (%)
**Age group (years)**					
	<50 (n=7)	3 (43)		4 (57)	
	50-64 (n=7)	1 (14)		6 (86)	
	≥65 (n=13)	4 (31)		9 (69)	
	Missing (n=2)	2 (100)		0 (0)	
**Sex**					
	Female (n=19)	5 (26)		14 (74)	
	Male (n=10)	5 (50)		5 (50)	

In addition, 80% (64/80) of the carers who consented to participate following general practice recruitment (group 2) completed the baseline survey (Table 2). Most (50/64, 78%) were female, 42% (27/64) were aged ≥65 years, 95% (61/64) were of White ethnicity, and 50% (32/64) had a higher education qualification. They reported a wide range of different caring responsibilities, with more than half (39/64, 61%) caring for someone who did not receive professional care; the mean time they reported as spent in caring was 6 days per week and 10 hours per day. In total, 33 (52%) of the 64 group-2 participants completed the follow-up survey.

**Table 2:** Characteristics of group-2 carers who completed the baseline survey (n=64).

Characteristics			Group-2 participants, n (%)
**Age group (years)**			
	<50	13 (20)	
	50-64	24 (38)	
	≥65	27 (42)	
**Sex**			
	Male	14 (22)	
	Female	50 (78)	
**Qualification**			
	General Certificate of Secondary Education or equivalent	18 (28)	
	Level A or equivalent	7 (11)	
	Higher education	32 (50)	
	Other	7 (11)	
**Employment status**			
	Full-time paid work	18 (28)	
	Part-time paid work	5 (8)	
	Retired	24 (38)	
	Looking after family or home	10 (16)	
	Other	6 (9)	
**Presence of any long-term health condition**			
	Yes	22 (34)	
**Ethnic group or background**			
	Ethnic minority group	3 (5)	
	White	61 (95)	

### Stakeholder Interviews

From 349 invitations to participate sent to relevant stakeholders (individuals and organizations), 52 (14.9%) expressed interest in being interviewed and 12 (3.4%) interviews were completed (Table 3). This included frontline workers and managers of related organizations from charities, local authorities, and health services. Recruitment occurred during the first year of the pandemic; it proved difficult to engage stakeholders’ interest in the study at a time when the health and social care systems were under considerable pressure.

**Table 3:** Organization types and role of stakeholders who were interviewed.

ID	Stakeholder role	Organization type
S-01	Manager	Dementia charity
S-02	Frontline worker	Community outreach
S-03	Manager	Health services
S-04	Frontline worker	Health services
S-05	Frontline worker	Community group
S-06	Manager	Cancer charity
S-07	Manager	Carers charity
S-08	Frontline worker	Secondary care
S-09	Manager	Social enterprise
S-10	Manager	Secondary care and community nursing provider
S-11	Frontline worker	Carers charity
S-12	Manager	Local authority

### Key Findings

#### Overview

From the integrated findings, seven overarching themes were identified that were associated with the reach, adoption, implementation, and maintenance of Care Companion:

1. Stakeholders’ signposting of carers to Care Companion
2. Expectations about Care Companion
3. Care Companion activity levels and conflicting priorities
4. Experience of using Care Companion
5. Relevance to personal circumstances
6. Social isolation and networks
7. Experience with digital technology

These are presented in the following sections with illustrative quotes for each theme: S indicates a stakeholder participant, C1 indicates a group-1 carer participant who registered with Care Companion directly, and C2 indicates a group-2 carer participant who registered following an invitation sent by their GP.

#### Stakeholders’ Signposting of Carers to Care Companion

Although several stakeholder participants described ways in which they had publicized the availability of Care Companion to carers in the area, such as through notices in their newsletters or by adding links to Care Companion through their website, only 1 of the interviewees had actively promoted its use as part of the service they provide to patients or clients. Others felt that it was inappropriate to “promote” Care Companion in preference to other available resources and apps:

We signpost to it for the benefit of the carers on our courses...So we don’t send out the link. The link is on the form...for them to read if they want to.

S-09

I send out newsletters to carers in Coventry and Warwickshire and I often feature some of the apps that are on there and Care Companion is one of the ones that I do promote...And that goes out to 4,600 carers in Coventry and nearly 2,000 carers in Warwickshire.

S-11

We do promote it at our health and wellbeing events which we have monthly...they should be getting a leaflet in their pre-assessment packs.

S-06

These promotional activities had led some carers to register with Care Companion:

I think [signpost to Care Companion] must have been from a Carers Trust thing.

C1-15

#### Expectations About Care Companion

Carers gave wide-ranging reasons for registering with Care Companion. There was a general expectation that Care Companion might help with the challenges associated with their current circumstances but often without a view about how this would happen:

It was suggested to me by a friend actually. They’d heard of it, they hadn’t actually used it, but...they said to me, “Do you know what, this might actually be really useful for you...Probably worth having a look at.”

C1-10

I thought it would probably be a good idea as a way to find out about it and to see if it would be useful to me and help my life be a bit more easier.

C1-12

Some were clear about how they expected Care Companion to help them address information needs that were condition specific or service related:

I was very conscious of the fact that when we come out of lockdown I need to know about local services and things and what’s...going on...and what’s on offer.

C1-13

Although Care Companion does not provide functionality to enable contact with other carers for peer support, some carers mistakenly expected that it would offer this benefit and help address feelings of being alone:

I really just wanted someone to talk to someone, you know, someone who understood.

C2-03

A stakeholder interviewee also misunderstood what Care Companion offers and thought that it allowed interaction with “care companions” through direct communication or on the web:

Oh, I think if the Care Companions have the training and the knowledge, and I’m sure they do, of the signs to look out for when a carer’s not so mentally well, and how they can support them...

S-10

#### Care Companion Activity Levels and Conflicting Priorities

As shown in Table 4, analysis of the web logs indicated that most carers who registered with Care Companion made little use of the resource. The mode and the median number of visits to the resource was 2, the median total number of actions (web pages clicked on, diary entries, etc) was 37, and the median total time spent on Care Companion was 26.7 minutes.

**Table 4:** Number of visits and actions and total time spent on Care Companion per registered user.

	Values, mean	Values, median (range)	Values, mode
Total number of visits/user	7.2	2 (1-125)	2
Total number of actions/user	85	37 (2-1479)	40
Total time spent on Care Companion (min)/user	75.5	26.7 (1.5-1210)	N/A^a^

^a^N/A: not applicable.

There were relatively high levels of engagement with the 6-week Care Companion email campaign that was automatically sent to users following their initial registration. These each focused on a specific topic related to *Getting the most out of Care Companion,* However, the opening rate for these emails dropped from 73% in week 1 to 54% by week 6, with the click rate (measuring interaction with an email) dropping from approximately 20% to <5%.

There was similar attenuation in the viewing of the YouTube tutorial videos that were associated with each of the engagement emails, from approximately 60 views each for the first videos that introduced how to use Care Companion to <10 views per video for latter ones that covered specific functions, suggesting diminishing interest over time.

From the interviews with carers, lack of time was a frequently described barrier to the use of Care Companion. Many carers attributed this to competing demands, whether at work or at home, in the context of already feeling that they were “at full stretch” and viewing Care Companion as something that involved a time investment:

If I’m honest I dipped in...I think the, the problem is because I’m working and I’ve got loads on...I really need to sort of sit down and set up, if that makes sense. And I haven’t really had time to do so.

C2-69

It takes time for me to invest in [Care Companion] by recording things or entering information, or putting details in the address book and things like that, that’s the biggest limitation to me.

C1-12

This view was echoed by a stakeholder who had heard it expressed by carers:

They might not have time to prioritise it and it’s not something which they feel is worth prioritising because it’s a, a, “Nice to have as a carer,” rather than a, “It’s going to provide me with immediate results now in this minute.”

S-03

#### Experience of Using Care Companion

The follow-up carer survey found that, of the 33 participants, only 7 (21%) described having used Care Companion within the previous 3 months, 4 (12%) felt that it had useful information, and 3 (9%) agreed that it had helped them cope with their role as a carer. The most frequently used functions within Care Companion were its diary, the resources section, and the help videos. Other parts (eg, mood monitor, directory of useful contacts, and frequently answered questions and glossary section) were rarely or never used. Of the 33 participants, 4 (12%) participants anticipated their use of Care Companion to increase in future, 11 (33%) anticipated that it would stay the same, and the remainder (18/33, 55%) anticipated a decline. Although 70% (19/27) of the participants agreed or were neutral about viewing Care Companion as relevant to their personal situation, most tended to agree or were neutral regarding with statements that they could find the information more easily elsewhere (22/28, 79%) and that Care Companion being time consuming to use (20/29, 69%).

Several stakeholders felt that Care Companion was relevant to supporting the needs of carers in terms of offering a supportive resource, accessible at any time and from anywhere:

I think it’s important to utilise, you know the online world that we have internet and all that...And, you know, you don’t necessarily have to go to, drive to, a class or, or you know, see a counsellor, or something like that.

S-13

I think there’s so much information and resources that carers are able to tap into, and I think because [Care Companion] helps with sort of looking at what’s available in their local community...that’s really important to the carers. As is having sort of diary functions on there, with the address book where they can sort of put in the information. I think that’s all really, really useful.

S-11

Some carers felt that there was a lack of breadth in the resources included, whereas others felt that it needed a more narrowly defined focus on the needs of a specific subgroup of carers:

My only complaint would be that everything is now coming from the same place...But there’s no other, you know, no other point of view and, and no other opinion.

C1-10

I think you need to narrow the focus down significantly...I mean, by trying to do everything, you’re doing everything badly, if I could be brutally honest...

C2-72

Some felt that Care Companion was most likely to be of value to those who were taking on new caring roles; however, some felt that it needed to offer a more directive, instructional approach:

If somebody is just starting to look after someone, you’re floundering about knowing which organisations – should it be social services, should it be health services, should it be a particular support group? So having a central website that you can log onto, would be very useful, and yes I would recommend it.

C2-73

I was hoping that there would be a bit more on, “I’m a new carer. What do I do?”...From my past experience, it helps if you’ve got like this first thing, roadmap or whatever you want to call it...

C1-15

#### Relevance to Personal Circumstances

Some carers described ways in which Care Companion had helped them access local services or find information that was relevant to their needs and their care recipients at times when this was needed:

There is loads and loads of information. And it’s all in one place which is good. And there’s links, isn’t there, so it goes off to other pages if you need them.

C1-09

I was having a really bad week, I thought, “Oh, I’m gonna have a look at Care Companion] and see what I can find.” And I came across, I think it was the Carers Team. Anyway, I contacted them, somebody rang me back...

C2-10

I did spend quite a lot of time [on Care Companion] researching things, sometimes for my own health, not just my mother’s health.

C1-15

However, other carers described difficulty in finding information that was directly relevant to the complex situation and challenges that they were facing:

There is a lot of information on there, and it is quite easy to navigate around, it’s just obviously knowing what bit you’re looking for. For me, that’s where I find it difficult...such a complex situation.

C2-101

I don’t think Care Companion can help me on that, because it’s really a very tricky thing, dementia.

C1-05

Several carers described how they already had established ways of managing their carer responsibilities (eg, using paper diaries, spreadsheets, and web-based search engines) and saw little added value from Care Companion. This view was also recognized by some stakeholders:

I keep like a proper address book anyway...And my diary tends to be written on the calendar or even, you know, occasionally I’ll, I’ll put stuff on the computer if it’s something that, that I need a definite reminder about.

C1-10

They already use their smartphone for example which allows them to collate some of that information already or they’ve already got an app...or they already use a hard copy journal or various different things and they don’t feel that [Care Companion] gives them anything extra.

S-03

Some features, although viewed as being valuable by stakeholders, were not felt by carers to be important. Potentially, they could be burdensome, and their usefulness, in terms of how this would help the carer and the person they cared for, was unclear:

The mood monitor [in Care Companion], I think that’s really beneficial for people. For the cared for and the carer. Particularly that it can help them to kind of highlight any patterns.

S-12

I’m not that interested in putting smiley faces [Care Companion’s mood monitor].

C2-19

#### Social Isolation and Networks

Both carer and stakeholder participants expressed disappointment that Care Companion does not tackle social isolation more directly as one of the most important issues for carers:

There’s a high percentage [of carers] that are socially isolated ‘cause they can’t leave the home. So they would physically benefit from having somebody come in and physically seeing them...I do know that they enjoy, speaking face-to-face is their preferred option.

S-11

I’m sure it’s good, I’m not criticising it...But I’m the sort of person who’d rather talk to someone, you know.

C2-29

However, participants recognized that Care Companion might enable access to social networks, by providing information about their availability and how to access them, and to services. This provided reassurance:

I can see they’ve got lists of contacts and things like that would be really good...things that I haven’t used yet, but I might use in the future...Kind of reassuring knowing it was there for the future. It’ll be on the day when I, I’m tearing my hair out that you...reach for it.

C2-105

Being unable to share appointments or have multiple carers on 1 care recipient profile was seen by some as limiting the usefulness of Care Companion:

I wouldn’t use it for appointments and things. Because I need to see my appointments and my husband’s appointments and my mum’s appointments, and my husband needs to see them as well.

C2-73

#### Experience With Digital Technology

From the baseline carers’ survey, when asked about their use of the internet in general, of the 33 participants, 28 (85%) reported using a smartphone daily, 26 (79%) checked emails daily, and 22 (67%) used the internet daily to check the news and weather, whereas 21 (64%) used internet searches daily. Approximately two-thirds (20/33, 61%) did weekly web-based shopping and one-third (10/33, 30%) were using apps and websites (not including Care Companion) to assist in their caring role.

Carer interviews identified differing levels of confidence in using digital technology, which in turn affected their view about Care Companion. For some, IT experience had developed considerably during the COVID-19 pandemic, for example, by obtaining a smartphone and becoming skilled at meeting family and friends via videoconference. Many already used search engines for information regarding services or particular health conditions related to their caring roles:

My daughter lives 300 miles in xxx, so I’ve bought a smartphone so I can WhatsApp her and message her. I use a computer for ordering food, you know. I wouldn’t say I’m, I’m very good, but I’ve got a smartphone and I can order stuff and, and WhatsApp my daughter.

C2-03

And also Zoom which a couple of months ago I’d never heard about or, well, I’ve heard about but never done anything with it, but I have Zoom meetings now nearly almost twice a week...[C1-05]

The benefit of using Care Companion instead of search engines to avoid the risk of being overwhelmed by links to websites of spurious quality was generally recognized; however, there were some carers who expressed being comfortable with using search engines:

...To have information] in one place is very good. ‘Cause as soon as you start, you put some of this stuff in Google, it just brings, brings a huge list out and...massive list. And also, you don’t know the quality of the sites that you’re looking at...

C2-99

And the resources are good. I like the resources. But...it’s trying to decide how are the resources different on Care Companion to what I can just Google.

C1-12

Some stakeholders expressed concerns that carers who lacked IT access or literacy would be unable to use Care Companion:

None of our clients or the people that we work with have been significantly interested in, in pursing it...common reasons include that they aren’t very technological savvy...[S-03]

There is that downside that there are those carers who can’t access [Care Companion] because...they don’t have the technology or up-to-date smartphones and things like this to be able to.

S-07

## Discussion

### Principal Findings

This study of the real-world use of Care Companion, a freely available web-based information resource codeveloped to address the needs of informal carers, found that in the first 3 years following its launch, uptake remained low. Only 476 carers (0.74% of the area’s known carer population of 100,000) registered to use it following general promotion via health care, social care, third-sector, and voluntary organizations and a further 80 (3.8% of the known population of 2105 carers in the participating practices) registered following invitations from GPs. Most registered users only logged into Care Companion once or twice. Although most stakeholder and carer participants identified potential value in Care Companion’s content, many felt that it was likely to be more relevant when first becoming a carer or when the care recipient’s needs were undergoing significant change.

Overall, 7 themes were identified, which affected carers' uptake and use of Care Companion. Key issues included mixed understanding of Care Companion’s purpose and content (both by carers and stakeholders); the lack of time to explore what Care Companion’s offers, reflecting conflicting carer priorities and concerns; perceived lack of relevance to current personal needs, such as social isolation and the need for hands-on support; and the perceived effort required to use Care Companion outweighing any expected benefit. Many carers felt that their existing coping strategies limited their immediate need for a resource of this type, and some believed that their current situation was very complex for Care Companion to be of benefit.

Although Care Companion was launched before the COVID-19 pandemic, data collection was undertaken at the time when social distancing, lockdown, and shielding restrictions were still in place. Although carers had to cope with extra demands, great isolation, and significantly great strain on their mental health [2,30,31], there was no evidence that this increased the interest in using Care Companion; instead, these extra demands may have limited the time and the privacy that carers had available to explore Care Companion.

Few stakeholder organizations appeared to have actively encouraged their frontline staff to promote the use of Care Companion to their carers. This may have reflected skepticism about the importance or value of providing an information resource and agnosticism over the endorsement of apps or web-based services in general. In addition, stakeholders had mixed understanding of Care Companion’s content and functions and concerns that the use of Care Companion might exacerbate inequalities, given the limited digital literacy and access to IT for some older carers [2].

The multidimensional focus of Care Companion reflected the priorities that emerged during its co-design [19], but there were widely divergent views expressed in this study about whether this was a strength or a limitation. Some carers viewed Care Companion as lacking focus and direction, especially for individuals who are new to caring roles, whereas for others, it was felt to lack relevance to the complexity of their caring needs and situation. This highlights the need for widely diverse views to be included in the co-design process, as this was an issue that had not emerged previously.

Although Care Companion could be used by the carer and care recipient together to support mutual dependency [32], its design did not facilitate such interaction. Furthermore, its profile could not accommodate the carer having >1 care recipient to care for or conditions where a couple were cocaring for each other. This may have limited perceptions about its usefulness. An element that was intended for shared use was the mood monitor that provided a means of recording carer and care recipient moods. However, this emerged as being one of the least used components of Care Companion, with few carers feeling that it was meaningful in the context of managing their carer and care recipient relationship.

At the time of this study, Care Companion was internet-based and not available as an app. Apps are generally experienced as being more convenient, faster, and easier to browse than websites [33]. Although Care Companion provided multiple functions, which emerged as a recommendation in a recent review of mobile apps for carers [34], not being available in an app format may have contributed to the time and difficulties involved in its use.

### Comparison With Previous Studies

Older adults’ willingness to adopt new technologies is most influenced by its perceived value, the perceived improvement in quality of life that might follow, and their confidence in being able to use it [35]. Although the co-design of Care Companion was intended to optimize its relevance and ease of use, the findings from this study indicate that carers had mixed views about the relevance of Care Companion, effort involved in its use, and likelihood of it having a significant impact on their caring role and quality of life, which contributed to the low level of use.

However, Care Companion is not unusual in its low levels of uptake among carers; multiple studies have reported low uptake rates for digital interventions and decreasing use over time [36,37]. As has been observed for other digital and telehealth interventions for carers, time and effort are key barriers to uptake and use, in addition to how they fit into carers’ current routines [38,39]. Care Companion was described by some carers as lacking sufficient relevance to their personal needs to merit the time and effort required to fully engage with it. Many carers felt that the task of setting up an alternative approach, such as that needed to use Care Companion’s diary function, would add to their problems rather than relieve them.

Care Companion was based on a transactional approach to the support of carers: it provided information and methods for organizing the day and keeping track of events and contacts. This was intended to help carers feel more in control, be better informed, and build resilience. However, recent evaluations of interventions, both digital and face to face, have emphasized the relational or emotional aspects of caring and the need for information provision and other supportive measures to acknowledge and take account of these in their methods of delivery [40,41]. Exploratory investigation adopting a *capabilities approach* has also highlighted the relational nature of caring, focusing on the value of the capability for caring in relation to other valued capabilities and their potential conflict [42].

A number of multiple-component interventions have found that facilitating interaction with professionals is more beneficial than information alone [43,44], with carers expressing frustration when required to review information that was not directly relevant to their specific needs [45,46]. The Europe-wide InformCare web platform, for example, also found that its information resources area was infrequently used but that its interactive services, social network, and private messaging, which addressed caregivers’ needs to communicate with others and share experiences, were more widely accessed [47].

A limitation of Care Companion perceived by several stakeholders and carers was that it did not directly provide a means of interaction with peers or professional support. During the co-design of Care Companion, consideration had been given to the inclusion of peer interaction through some kind of forum, but it was decided that rather than replicating the availability of several established web-based forums, Care Companion should promote awareness of such forums and support groups through its resources section. The benefits of web-based peer networks, either alone or as an element within a broad intervention, have been demonstrated [36,48]. However, studies delivering multicomponent programs that included unstructured support by professionals and peers did not show significant changes in psychological outcomes [49,50],

### Strengths and Limitations

A key methodological strength of this study is that it involved a mixed methods exploration of real-world patterns of use and the reasons underlying this. The study drew on a wide range of quantitative and qualitative data sources to describe what happens when a resource such as Care Companion is made available to the carer population without any requirement for carers to commit to using it in a particular way or within a specific time frame. However, the comparatively low levels of registration in and use of Care Companion severely limited the extent to which meaningful quantitative and qualitative analyses could be undertaken. However, the overarching themes that emerged from the convergent data synthesis enabled a broad representation of the reach, adoption, and use of Care Companion.

The interviews with carers and stakeholders allowed a range of perspectives to be identified and provided insights into the possible facilitators of and barriers to the uptake and use of Care Companion. In addition, there was regular patient and public involvement throughout the study, which aimed to ensure that the design, data collection, and interpretation of findings reflected the priorities of carers. However, the study was limited to carers who had registered with Care Companion, and hence, it was beyond its scope to evaluate why carers did not register. The experiences and views described by the study participants who registered and then made little use of Care Companion are likely to overlap with those of carers who chose not to register at all; there may have been other reasons that contributed to carers not registering with Care Companion that we failed to identify.

The COVID-19 pandemic added to the difficulties of promoting interest in Care Companion among stakeholders and carer groups. Carer groups completely stopped meeting or attempted to meet on the web during the pandemic. This may have adversely affected registration with the resource and participation in the study. For example, the recruitment of stakeholders occurred during the last few months of 2020, a time during the COVID-19 pandemic when many stakeholders were working from home and difficult to reach or had been furloughed and when involvement in research may not have been viewed as a priority.

### Conclusions

This evaluation of Care Companion found a very low level of uptake and use following an area-wide launch and signposting to carers by stakeholder organizations. The gap between the views of carers and stakeholders expressed during the co-design and user acceptance testing [19,20], with the subsequent real-world experience following its launch, raises 2 issues. The first is about the inclusivity and diversity of the carers and stakeholders participating in the co-design and the extent to which their views were heard and reflected in the development and implementation of Care Companion. Inevitably, carers and stakeholders who volunteer to participate in a co-design process are likely to be more interested and committed to its intended outcome than their peers. This highlights the importance of actively seeking as diverse a range of viewpoints as possible during intervention co-design: more rigorous testing of the design with the target population before proceeding with its development may then have seen more of the 476 people who registered with Care Companion make significant use of it. However, when introducing an innovation, there is only so much that can be learned about users’ requirements before they have the opportunity to use it in practice [51]. The second issue is about the provision of support after deployment that will enable an innovation to evolve alongside users’ emerging requirements [52,53]. The importance of designing effective, interactive, and dynamic ways of addressing carers’ complex and varied information needs as a key part of their support remains as an issue.

## Abbreviations

GP: general practitioner
RE-AIM: Reach, Effectiveness, Adoption, Implementation, and Maintenance

## Data Availability

The data sets generated and analyzed during this study are available from the corresponding author upon reasonable request.
